# Association of metabolic‐associated fatty liver disease and risk of severe coronavirus disease 2019 illness

**DOI:** 10.1002/jgh3.12465

**Published:** 2020-12-01

**Authors:** Marianne Linley Sy‐Janairo, Ian Homer Y Cua

**Affiliations:** ^1^ Institute of Digestive and Liver Diseases St. Luke's Medical Center Global City Taguig Philippines

**Keywords:** COVID‐19, fatty liver, non‐alcoholic fatty liver disease, SARS‐COV2

## Abstract

Given the global health burden caused by the Coronavirus Disease 2019 (COVID‐19), there have been numerous studies aimed to understand its clinical course and to determine risk factors that may impact prognosis. Pre‐existing medical conditions are linked with COVID‐19 severity, particularly cardiometabolic diseases. Increasing evidence has also linked metabolic‐associated fatty liver disease (MAFLD) with severe COVID‐19 illness. Thus, we review different published clinical data relating to the association of MAFLD and COVID‐19 severity. Our review showed that published studies consistently support the association between MAFLD and more severe COVID‐19, even after adjustment for confounding factors. It was also observed that an increasing hepatic fibrosis score is correlated with increasing severity of COVID‐19. Finally, younger age and obesity among MAFLD patients also led to a greater risk of severe illness.

## Graphical abstract

Published studies consistently support the association between metabolic‐associated fatty liver disease and more severe COVID‐19.

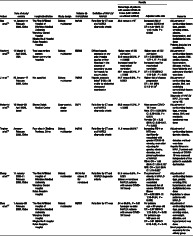

## Introduction

The coronavirus disease 2019 (COVID‐19), caused by the severe acute respiratory syndrome coronavirus 2 (SARS‐CoV2), has affected millions of lives globally. The outbreak was first reported in Wuhan City, Hubei Province, China at the end of 2019. The infection has spread globally and has now been reported in >180 countries. In March 2020, the World Health Organization (WHO) declared COVID‐19 a pandemic,^1^ which continues to claim thousands of lives every day.

The spectrum of COVID‐19 illness severity ranges from asymptomatic disease to critical illness. The study of the course and outcomes of COVID‐19 from the Chinese Center for Disease Control and Prevention involving 44 415 COVID‐19 patients reported that a majority have mild disease (81% of cases).^2^ Around 14% of cases develop severe illness requiring hospitalization and oxygen support, while 5% are critically ill patients requiring intensive care support.^2^ The case fatality rate is 49% among the critical cases, where a total of 1023 deaths were documented out of the 2087 critically ill patients.^2^ All of the mortality in this study occurred in this group of patients.

Given the impact of disease severity on prognosis, there has been much interest in identifying risk factors associated with severe COVID‐19. Increasing age has been consistently associated with severe infection and higher mortality.^2^, ^3^, ^4^, ^5^ Although any age group is susceptible to the virus, older patients are more likely to be hospitalized, have severe illness, and a higher risk of death.^2^, ^3^, ^4^ Other established risk factors for severe illness include pre‐existing lung disease, chronic kidney disease, diabetes mellitus (DM), hypertension, cardiovascular disease, and obesity.^2^, ^6^, ^7^, ^8^ Chronic liver disease is another comorbid condition that is being evaluated for its impact on COVID‐19 severity. A multicenter study conducted in 13 Asian countries has demonstrated that pre‐existing liver disease is an additional risk factor for having severe COVID‐19 illness, in which the outcomes are correlated with the patients’ hepatic reserve and liver synthetic functions.^9^ The findings were in agreement with the study of Hashemi *et al*., which found that patients with chronic liver disease have more critical illness.^10^

One of the most common liver diseases worldwide is metabolic‐associated fatty liver disease (MAFLD), previously known as non‐alcoholic fatty liver disease (NAFLD). Studies have suggested its possible association with severe COVID‐19 illness. In the study of Ji *et al*.,^11^ patients with fatty liver had a higher risk of COVID‐19 disease progression, a greater likelihood of abnormal liver tests from admission to discharge, and longer virus shedding time. The exact mechanisms through which it contributes to a more severe COVID‐19 illness remains unknown.^12^ In this review, we provide a consolidated update on the current available evidence related to the association of MAFLD and severe COVID‐19 illness.

## Methods

PubMed, Embase, and Google Scholar databases were searched for relevant articles from November 1, 2 019 to August 31, 2020 using the search terms “NAFLD” or “Non‐alcoholic fatty liver disease” or “MAFLD” or “Metabolic associated fatty liver disease” AND “COVID‐19.” Bibliographies were reviewed for articles that could qualify for the review. Inclusion criteria for the selection of studies were: (i) reported association between MAFLD/NAFLD and COVID‐19 severity; (ii) randomized controlled, case–control, or cohort design trials; and (iii) study population comprising adult patients (≥18 years).

The main outcome of interest is the association of MAFLD and severity of COVID‐19 illness. If an article involved chronic liver disease, the study was included if the information regarding MAFLD and its association with COVID‐19 illness severity was available. Odds ratio (OR) was used if available. When both the crude and the adjusted OR values were provided, the adjusted value was adopted. If only the raw data were reported, the unadjusted OR was calculated to ensure uniformity of reporting results among the included studies. The percentage of MAFLD *versus* non‐MAFLD patients with severe COVID‐19 illness was also presented if provided by the study.

## Discussion

The impact of pre‐exiting liver disease such as MAFLD on COVID‐19 illness is now being evaluated. An international expert consensus defined MAFLD as the presence of hepatic steatosis among individuals that meet at least one of the following conditions: overweight/obese, presence of type 2 DM, or evidence of metabolic dysregulation.^13^ *Table* 1 shows the new positive diagnostic criteria for MAFLD among adults. Compared to the previous definition of NAFLD, exclusion of excessive alcohol consumption or other causes of chronic liver disease is not mandatory to diagnose MAFLD. Instead, patients with other concomitant conditions are defined as having dual or more etiologies of fatty liver disease.

**Table 1:** Diagnostic criteria for metabolic‐associated fatty liver disease (MAFLD) in adults (Adapted from Eslam *et al*.^13^ with permission.)

Components	Clinical definition/clinical cut‐off values
Hepatic steatosis	Detected either by liver histology, imaging techniques, or blood biomarkers/scores
AND	
At least *one* of the following:	
Type II diabetes mellitus	According to widely accepted international diagnostic criteria for type II diabetes mellitus
Overweight or obesity	Caucasians: BMI ≥25 kg/m^2^ Asians: BMI ≥23 kg/m^2^
Lean/normal weight but with evidence of metabolic dysregulation	Caucasians: BMI <25 kg/m^2^ Asians: BMI <23 kg/m^2^ AND At least *two* of the following: Waist circumference: Caucasian: Men ≥102 cm, women ≥88 cm Asian: Men ≥90, women ≥80 cm Blood pressure ≥130/85 mmHg or specific drug treatment Plasma triglycerides ≥150 mg/dL (≥1.70 mmol/l) or specific drug treatment Plasma HDL‐cholesterol: Men <40 mg/dL (<1.0 mmol/L) Women <50 mg/dL (<1.3 mmol/L) or specific drug treatment Prediabetes (i.e. fasting glucose levels 100–125 mg/dL (5.6–6.9 mmol/L), or 2‐h postload glucose levels 140–199 mg/dL (7.8–11.0 mmol) or HbA1c 5.7–6.4% (39–47 mmol/mol) Homeostasis model assessment (HOMA)‐insulin resistance score ≥2.5 Plasma high‐sensitivity C‐reactive protein (hs‐CRP) level >2 mg/L

BMI, body mass index.

### *Proportion of severe* *COVID‐19 patients with and without* *MAFLD*

The proportion of patients with severe COVID‐19 illness is higher among patients with MAFLD compared to patients without MAFLD (11.6–50.9% *vs* 6.6–35.2%) across all of the pooled studies (*Table* 2). This provides a percentage difference between the two groups of 5–15.7%. Five studies^10^, ^11^, ^12^, ^14^, ^15^ directly assessed the association of MAFLD and severe COVID‐19 illness. A significant association between MAFLD and COVID‐19 severity was consistently evident in all included studies, except for the elderly group of patients in the study of Zhou *et al*., as shown in *Table* 2. The OR in these studies ranged from 1.10 to 31.2. This wide confidence interval reflects poor accuracy of the obtained association.

**Table 2:** Characteristics and findings of included studies

Author	Date of study/country	Hospital/institution	Study design	Patients (*n*) NAFLD/total	Definition of NAFLD/MAFLD	Results		Remarks
						Percentage of patients with severe COVID‐19 illness (with MAFLD *vs* without MAFLD)	Adjusted odds ratio	
Gao *et al*.^14^	17 January–11 February 2020, China	The First Affiliated Hospital of Wenzhou Medical University Wenzhou Central Hospital Ningbo No.2 Hospital Ruian People's Hospital	Cohort; multicenter	65/130	Fatty liver by CT scan and MAFLD diagnostic criteria	26.2 *versus* 7.7%^†^	Increased risk of severe COVID‐19 OR = 4.07 (95% CI 1.10–15.09, *P* = 0.025)	Adjustment of confounding factors (age, gender, smoking status, obesity, hypertension, and dyslipidemia) was done Patients included are nondiabetic.
Hashemi *et al*.^10^	11 March–2 April 2020, USA	Two large tertiary centers Seven community hospitals	Cohort; multicenter	55/363	Diffuse hepatic steatosis on any prior imaging studies or liver histology in the absence of secondary causes for hepatic fat accumulation	Higher rates of ICU admission 50.9 *versus* 35.2%, *P* = 0.0095 Increased need for mechanical ventilation 49.1 *versus* 30.4%, *P* = 0.006	Higher rates of ICU admission OR = 2.30 (95%CI 1.27–4.17. *P* = 0.03) Increased need for mechanical ventilation OR = 2.15 (95%CI 1.18–3.91. *P* = 0.02)	Adjustment of confounding factors (age, gender, BMI, cardiac disease, hypertension, diabetes mellitus, hyperlipidemia and pulmonary disorder) was done
Ji *et al*.^11^	20 January–17 February 2020, China	Not specified	Cohort; multicenter	76/202	Hepatic steatosis index^‡^ (HSI) > 36 and/or by abdominal ultrasound examination	44.7 *versus* 6.6%, *P* < 0.0001	Higher risk of disease progression OR = 6.4 (95% CI 1.5–31.2)^†^	Adjustment of confounding (age, gender, BMI, and underlying comorbidity) factors was done Diagnosis of NAFLD/MAFLD is made using HIS.
Mahamid *et al*.^15^	15 March–30 April 2020, Israel	Sharee Zedek Medical Center	Case–control; single center	22/71	Fatty liver by CT scan and MAFLD diagnostic criteria	36.3 *versus* 10.2%, *P* < 0.005	More severe COVID‐19 illness Male: OR = 3.29 (95% CI: 3.28–3.58, *P* = 0.001) Female: OR = 3.25 (95% CI: 3.09–3.47, *P* = 0.002)	Adjustment of confounding factors (age, smoking status, BMI, and metabolic syndrome) was done
Targher *et al*.^12^	January–February 2020, China	Four sites in Zhejiang Province, China	Cohort; multicenter	94/310	Fatty liver by CT scan and MAFLD diagnostic criteria	11.6 *versus* 26.5%^†^	Increasing FIB‐4 or NFS was significantly associated with greater COVID‐19 severity, even after adjusting for gender, obesity, diabetes, and presence/absence of MAFLD FIB‐4: OR = 1.90 (95% CI 1.33–2.72)^†^ NFS: OR = 2.57 (95% CI 1.73–3.82)^†^	Adjustment of confounding factors (gender, obesity, diabetes mellitus) was done Hepatic fibrosis in MAFLD patient and severity of COVID‐19 was assessed independent of the patient's metabolic dysfunction
Zheng *et al*.^16^	17 January 2020–11 February 2020, China	The First Affiliated Hospital of Wenzhou Medical University Wenzhou Central Hospital Ruian People's Hospital	Cohort; multicenter	66 (45 for obese; 21 for nonobese)	Fatty liver by CT and MAFLD diagnostic criteria	37.5 *versus* 9.5%, *P* = 0.021 (obese *vs* nonobese MAFLD patients with severe COVID‐19 illness)	The presence of obesity in MAFLD patients was associated increased risk of severe COVID‐19 illness OR = 6.32 (95% CI 1.16–34.54, *P* = 0.033)	Adjustment of confounding factors (age, gender, smoking, DM, hypertension, and dyslipidemia) was done Population is obese patients with MAFLD
Zhou *et al*.^17^	1 January–29 February 2020, China	The First Affiliated Hospital of Wenzhou Medical University Wenzhou Central Hospital Ningbo No.2 Hospital Ruian People's Hospital	Cohort; multicenter	93/327	Fatty liver by CT scan	24 *vs* 55.6%, *P* = 0.01(younger *vs* older MAFLD patients with severe COVID‐19 illness)	For patients age < 60 years old OR = 2.67 (95%CI 1.13–6.34, *P* = 0.03) For patients age ≥60 years old OR = 0.61 (95%CI 0.18–2.03, *P* = 0.42)	Adjustment of confounding factors (age, gender, smoking status, overweight, diabetes, and hypertension) was done Small population size, especially for the elderly group

^†^ *P* value was not reported.

^‡^ HSI = 8 × [ALT/ AST] + BMI [+ 2 if type 2 diabetes yes, + 2 if female.

BMI, body mass index; COVID‐19, coronavirus disease‐19; CT, computed tomography; FIB‐4, Fibrosis‐4; MAFLD, metabolic associated fatty liver disease; NAFLD, non‐alcoholic fatty liver disease; NFS, NAFLD fibrosis score; OR, odds ratio.

The highest OR was seen in the study of Ji *et al*.^11^ Compared with the other studies that used computed tomography scan and/or MAFLD diagnostic criteria for the identification of included patients, Ji *et al*. utilized the hepatic steatosis index and/or abdominal ultrasound. The difference in the diagnostic accuracy of the method used for identifying the patients with MAFLD may have an impact on the results of the study. Thus, this may be a possible source of heterogeneity. When this study was excluded, the new OR across the four studies ranged from 1.10 to 15.09. The narrower confidence interval may reflect a more accurate estimate of the association.

### *Risk factors of severe* *COVID* *among* *MAFLD* *patients*

Patients with MAFLD often have coexisting medical conditions that may contribute to severe COVID‐19 illness. Increasing age, obesity, and presence of hypertension and DM are risk factors for severe COVID‐19. With the current evidence, it is difficult to establish whether the association between MAFLD and COVID‐19 severity is secondary to these factors, despite the adjustments for confounders in the different studies. Whether the impact of these confounding factors is different among patients with MAFLD is also uncertain.

Older age is considered to be a clinical predictor of fatal outcome in COVID‐19.^2^, ^3^, ^4^, ^5^, ^7^, ^8^ Elderly patients tend to have more severe and chronic disease, contributing to poor prognosis. In the study of Zhou *et al*.,^17^ MAFLD patients were divided into younger and older age groups. A synergistic effect of age and MAFLD was found for severe COVID‐19 among younger patients (aged younger than 60 years), even after adjustment for confounding factors such as age, gender, smoking status, overweight, diabetes, and hypertension. This association was not seen in the elderly group (OR = 0.61, 95% confidence interval [CI] 0.18–2.03). However, it is important to note that the elderly group had a small population in this study. Thus, additional studies and a larger sample size are recommended to make a more definitive conclusion. The reason for this finding is not well established but could be attributed to differences in hepatic and systemic immune responses caused by MAFLD in the different age groups.^17^ A reply letter by Ji and Qi *et al*. postulated that younger patients with MAFLD might have more hepatic inflammation, leading to more severe COVID‐19 illness.^18^ Furthermore, when data for both age groups in the study of Zhou *et al*. were combined, fatty liver disease remained an independent risk factor for severe COVID‐19 illness.^18^

The study of Zheng *et al*.^16^ showed that obesity among MAFLD patients was associated with a sixfold increase (OR = 6.32, 95% CI 1.16–34.54, *P* = 0.033) in the association with severe COVID‐19, even after adjustment for confounding factors such as age, gender, smoking, diabetes, hypertension, and dyslipidemia. The risk of obesity among patients with MAFLD was found to be greater than those without MAFLD. Although obese patients are inherently considered to be at high risk for severe COVID illness,^19^, ^20^ having MAFLD may aggravate this risk. Increased visceral fat is directly associated with liver inflammation and fibrosis,^21^ and this might lead to an exacerbated cytokine storm among COVID‐19 patients.

Another metabolic disease related to MAFLD is DM. Type II DM was found to be associated with increased risk for severe COVID‐19 illness and mortality.^22^, ^23^ The proportion of severe COVID‐19 illness increased progressively in relation to glucose abnormalities at admission,^22^ and a well‐controlled blood glucose level correlated with improved outcome.^23^ To our knowledge, there is still no published study that has evaluated the association of DM among MAFLD patients with the severity of COVID‐19 illness. The incidence of DM among patients with NAFLD was found to be 11.6%, with no statistically significant difference between its incidence among patients with and without NAFLD.^24^

Interestingly, the study of Targher *et al*.^12^ evaluated the effect of increasing hepatic fibrosis in relation to the severity of COVID‐19 illness. Noninvasive fibrosis scores, such as the Fibrosis‐4 (FIB‐4) index and NAFLD Fibrosis Score (NFS), were used to categorize the degree of hepatic fibrosis in this study. A significant association was seen with increasing hepatic fibrosis scores and severe COVID‐19 illness, even after adjusting for gender, obesity, diabetes, and presence/absence of MAFLD. Data from this study suggest the possible contribution of advanced hepatic fibrosis to severe COVID‐19 illness, irrespective of the presence MAFLD. However, whether the calculated scores are valid to assess fibrosis stage in those with an acute, potentially severe illness has not been demonstrated in the study.

### *Proposed pathophysiology for increased severity of* *COVID‐19 illness among* *MAFLD* *patients*

The exact pathophysiology on how MAFLD contributes to a more severe COVID‐19 illness is unknown, but several mechanisms have been postulated. Furthermore, most patients with MAFLD have coexisting metabolic disorders, which may be a risk factor for severe COVID‐19 illness.

Angiotensin II (ACE II) receptors used by the SARS‐CoV‐2 virus for cell entry are found to be expressed in hepatocytes and cholangiocytes.^25^ The expression of these receptors is higher among patients with chronic liver injury.^26^ Whether this increased expression of ACE II receptors results in a higher viral load and more severe disease is currently unknown. It should be noted that a study by Biquard *et al*. demonstrated that patients with MAFLD have no changes in the liver expression of genes implicated in SARS‐CoV‐2 infection.^27^ It is therefore difficult to ascertain if MAFLD patients have higher viral loads.

Systemic inflammatory response syndrome, particularly the cytokine storm, is one of the pathophysiologic mechanisms for severe illness development in COVID‐19.^28^ Whether the elevated cytokine profile of patients with MAFLD renders them more vulnerable to severe COVID‐19 is also uncertain.^29^ Furthermore, the increased production of pro‐inflammatory cytokines among MAFLD patients may have contributed to this elevation.

### *Management implications*

As this review consistently shows the increased risk of MAFLD patients developing severe COVID‐19 illness based on currently available evidence, certain recommendations in terms of management of patients can be made. It is prudent to recommend closer monitoring of MAFLD patients with COVID‐19 illness, especially patients with comorbid conditions and other risk factors for more severe illness. The Asia‐Pacific position statement regarding the management of patients with liver derangement during the COVID‐19 pandemic recommends monitoring and timely adjustment of medications for metabolic disorders such as DM and hypertension.^30^ Monitoring and managing blood pressure and glucose level are likely to reduce the risk of adverse outcomes.^30^

### *Scope and limitation of the study*

A total of seven studies were included and reviewed. One of the studies^16^ dealt with obesity as a risk factor for severe COVID‐19 among patients with MAFLD. Another study^17^ stratified MAFLD patients according to age (younger *vs* older patients) in relation to COVID‐19 severity. The impact of chronic liver disease on outcomes of COVID‐19 infection was evaluated in one study^10^ and provided data regarding the association of MAFLD and outcomes of COVID‐19 illness. Six of the seven studies assessed COVID‐19 severity according to the protocol for novel Coronavirus pneumonia released by the National Health Commission & State Administration of Traditional Chinese Medicine (*Table* 3).^31^ The measurements of disease severity in the study of Hashemi *et al*. included mean hospital length of stay, prevalence of intensive care unit admission, rate of need for mechanical ventilation, and all‐cause in‐hospital mortality.^10^

**Table 3:** Clinical classification of COVID 19 severity for adults (adapted from the National Health Commission and National Health Commission and State Administration of Traditional Chinese Medicine)

Category	Clinical definition
Mild cases	Mild clinical symptoms, and No sign of pneumonia on imaging
Moderate cases	With fever and respiratory symptoms, and Radiological findings of pneumonia
Severe cases	Any of the following: Respiratory distress (Respiratory rate of ≥30 breaths/min) Oxygen saturation ≤ 93% at rest Arterial partial pressure of oxygen (PaO_2_)/Fraction of inspired oxygen (FiO_2_) ≤ 300 mmHg (1 mmHg = 0.133 kPa). In high‐altitude areas (at an altitude of over 1000 meters above the sea level), PaO_2_/FiO_2_ shall be corrected by the following formula:PaO_2_ = FiO_2_ × [atmospheric pressure (mmHg) = 760] Cases with chest imaging that shows obvious lesion progression within 24–48 h > 50% shall be managed as severe cases
Critical cases	Any of the following: Respiratory failure and requiring mechanical ventilation Shock With other organ failure that requires ICU care

*Table* 2 summarizes the characteristics of each included study. Six studies had a multicenter, cohort study design, while the remaining study had a single‐center case–control study design. Most of the studies (five of seven) were conducted in China. The other two studies were conducted in the United States and Israel. All of the studies adjusted for the impact of confounders when assessing the association between MAFLD and COVID‐19 severity. One of the studies (Hashemi *et al*.)^10^ stated the use of histologic diagnosis for the identification of fatty liver as a criterion for inclusion of patients but did not specify whether any of the included patients underwent liver biopsy for the diagnosis of MAFLD. The study of Ji *et al*.^11^ used hepatic steatosis index and/or abdominal ultrasound, while the others utilized computed tomography scan findings of hepatic steatosis along with the proposed diagnostic criteria for MAFLD.

One major concern seen among the included studies is the possibility of huge patient overlap as most studies were conducted in China. The study of Ji *et al*.^11^ and Targher *et al*.^12^ did not specify the hospitals in which their clinical data were obtained, while the others have essentially the same databases. Because of this, there is a high chance that the larger studies may include patients from all the smaller ones. Conducting more studies in other countries is recommended to generate a more accurate estimate of the association between MAFLD and severe COVID‐19 illness. This will also allow future review to generate a pooled OR across the different studies.

In addition, this review is limited by the nature of the included studies. The studies were retrospective in nature and mostly involved Asian populations. Only two studies were conducted in other countries aside from China, making the applicability of the results to other ethnic groups uncertain. As the studies did not have information regarding liver biopsy, we could also not assess the relationship to liver histology of MAFLD with COVID‐19 severity.

## Conclusion and perspective

A significant association was observed between MAFLD and COVID‐19 severity, regardless of coexisting medical conditions such as DM, overweight/obesity, hypertension, and dyslipidemia. Obesity increased the risk of severe COVID‐19 illness in this population. Furthermore, increasing noninvasive fibrosis scores were correlated with a higher risk for severe COVID‐19 illness.
